# Genomes and gene expression across light and productivity gradients in eastern subtropical Pacific microbial communities

**DOI:** 10.1038/ismej.2014.198

**Published:** 2014-10-21

**Authors:** Chris L Dupont, John P McCrow, Ruben Valas, Ahmed Moustafa, Nathan Walworth, Ursula Goodenough, Robyn Roth, Shane L Hogle, Jing Bai, Zackary I Johnson, Elizabeth Mann, Brian Palenik, Katherine A Barbeau, J Craig Venter, Andrew E Allen

**Affiliations:** 1 Microbial and Environmental Genomics Group, J. Craig Venter Institute, La Jolla, CA, USA; 2 Department of Biology and Biotechnology Graduate Program, American University in Cairo, New Cairo, Egypt; 3 Department of Biology, Washington University, St Louis, MO, USA; 4 Scripps Institution of Oceanography, University of California, San Diego, La Jolla, CA, USA; 5 Marine Laboratory, Nicholas School of the Environment, Beaufort, NC, USA; 6 Biology Department, Duke University, Durham, NC, USA; 7 Skidaway Institute of Oceanography, Savannah, GA, USA

## Abstract

Transitions in community genomic features and biogeochemical processes were examined in surface and subsurface chlorophyll maximum (SCM) microbial communities across a trophic gradient from mesotrophic waters near San Diego, California to the oligotrophic Pacific. Transect end points contrasted in thermocline depth, rates of nitrogen and CO_2_ uptake, new production and SCM light intensity. Relative to surface waters, bacterial SCM communities displayed greater genetic diversity and enrichment in putative sulfur oxidizers, multiple actinomycetes, low-light-adapted *Prochlorococcus* and cell-associated viruses. Metagenomic coverage was not correlated with transcriptional activity for several key taxa within Bacteria. Low-light-adapted *Prochlorococcus*, *Synechococcus*, and low abundance gamma-proteobacteria enriched in the>3.0-μm size fraction contributed disproportionally to global transcription. The abundance of these groups also correlated with community functions, such as primary production or nitrate uptake. In contrast, many of the most abundant bacterioplankton, including SAR11, SAR86, SAR112 and high-light-adapted *Prochlorococcus*, exhibited low levels of transcriptional activity and were uncorrelated with rate processes. Eukaryotes such as Haptophytes and non-photosynthetic Aveolates were prevalent in surface samples while Mamielles and Pelagophytes dominated the SCM. Metatranscriptomes generated with ribosomal RNA-depleted mRNA (total mRNA) coupled to *in vitro* polyadenylation compared with polyA-enriched mRNA revealed a trade-off in detection eukaryotic organelle and eukaryotic nuclear origin transcripts, respectively. Gene expression profiles of SCM eukaryote populations, highly similar in sequence identity to the model pelagophyte *Pelagomonas sp.* CCMP1756, suggest that pelagophytes are responsible for a majority of nitrate assimilation within the SCM.

## Introduction

The southernmost line of the California Cooperative Oceanic Fisheries Investigations (CalCOFI, calcofi.ucsd.edu) sampling grid encompasses significant physical, chemical and biological gradients. The innermost stations on the transect impinge upon the ‘green ribbon', a band of internal-wave-driven high primary productivity on the narrow continental shelf (Lucas *et al.*, 2011), while the outermost are typical of the oligotrophic subtropical Pacific. Therefore this transect (CalCOFI line 93) crosses major surface ocean biomes known to have different microbial community structure (Collier and Palenik, 2003; Zwirglmaier *et al.*, 2008; Allen *et al.*, 2012; Dupont *et al.*, 2012; Morris *et al.*, 2012) and dominant phylotypes. Additionally, along this transect a maximum in the pigment chlorophyll *a* (chl *a*), or subsurface chl *a* maximum (SCM), often forms at the thermocline, a sharp subsurface gradient in temperature, density and nutrient concentrations. Surveys of ribosomal RNA (rRNA) reveal that low-light-adapted phylotypes within the ubiquitous cyanobacteria lineage *Prochlorococcus* dominate at the SCM, while high-light-adapted phylotypes colonize the surface (Johnson *et al.*, 2006). Depth-dependent distributions of phylotypes are also observed for *Synechococcus* (Toledo and Palenik, 2003) and SAR11 (Vergin et al., 2013), though the connections to physiological adaptation remain obscure.

In most traditional oceanographic measurements of function (for example, primary production) or biomass (for example, particulate carbon (PC)), the community is integrated, precluding an assessment of the contribution from specific phylogenetic units. Shotgun sequencing-based metagenomics involves the random sequencing of genome fragments in a sample. This technique and other DNA-based methods such as quantitative-PCR of specific genes catalog the distribution and diversity of microbes and genes in the marine environment (Johnson *et al.*, 2006; Rusch *et al.*, 2007) but do not indicate activity. Metatranscriptomics involves the sequencing of community RNA after conversion to cDNA, providing information on both phylogenetic affinity and function for a subsampling of all of the genes being expressed by a microbial community. When coupled with rate measurements, this may facilitate assignment of phylotype to function. Although limited in number, metagenomic surveys of the Mediterranean SCM revealed a different percentage of guanine and cytosine profile from surface communities (Ghai *et al.*, 2010). The SCM of station ALOHA in the oligotrophic Pacific exhibits a divergent phylogenetic and functional profile relative to overlying surface waters (DeLong *et al.*, 2006). Metagenomics and metatranscriptomics have been coupled to study microbial communities in stratified water columns in the open ocean and coastal oxygen minimum zones, and they have highlighted an uncoupling between genomic enrichment and gene expression (Shi *et al.*, 2011; Stewart *et al.*, 2012). To date, such studies utilized prefiltration, thus excluding the large or particle attached prokaryotes, as well the majority of eukaryotes.

As part of the Global Ocean Sampling project (Rusch *et al.*, 2007), we combined measurements of community biomass, primary production and nitrogen uptake with metagenomics and metatranscriptomics to examine prokaryotic and eukaryotic diversity and function in the surface ocean and the SCM at four locations on CalCOFI line 93. The intersection of these data sets facilitated a preliminary breakdown of bulk ecosystem characteristics into defined taxonomic groups, identified active and less active microbial guilds, highlighted the previously unknown contribution of some taxonomic groups such as the Pelagophytes to nitrate reduction and demonstrated the utility of ecological genomics of single species (genome autoecology) as an indicator of environmental trophic state.

## Methods

### Oceanographic sampling

Water was collected with Niskin bottles mounted on a CTD rosette with an integrated chl *a* fluorometer (Turner Scufa, Sunnyvale, CA, USA), oxygen sensor (Sea-Bird Electronics, Bellevue, WA, USA) and nitrate sensor (Satlantic ISUS V1, Halifax, NS, Canada). ‘Surface' samples were obatined from 3 m, while chl *a* fluorescence maxima were targeted for SCM samples. All metagenomic and metatranscriptomic samples were collected at 1200 hours local time to minimize light effects. For metagenomic sampling, 200 l of seawater was passed through a 20-μm nytex mesh into 50 l carboys cleaned with 0.1% bleach and distilled water. The 20-μm-filtered seawater was serially filtered through 3.0-, 0.8- and 0.1-μm filters (Millipore, Billerica, MA, USA), with rapid transfer of filters to storage buffer and at −80 °C as described in Rusch *et al.* (2007). Nucleic acids were extracted as previously described (Rusch *et al.*, 2007) and sequenced using the Life Technologies pyrosequencing platform (454 Life Sciences, Branford, CT, USA). Metagenomic and metatranscriptomic data has been deposited at the CAMERA Data Distribution Center (DDC) under Project Name, Southern California Bight microbial metagenomes and accession ID: CAM_P_0001069. Nitrogen uptake and primary production measurement methods are described in the Supplementary Materials.

For metatranscriptomic sampling, 20-μm-filtered seawater was passed through a 0.2-μm sterivex filter for 30 min (typically 1.5–2 l), after which the sterivex was capped, flash frozen in liquid nitrogen and transferred to −80 °C. RNA was purified from filters using Trizol reagent (Life Technologies), treated with DNase (Qiagen, Valencia, CA, USA) and cleaned with the RNeasy MinElute Kit (Qiagen). For total mRNA transcriptomes, 250 ng of total community RNA was used for subtractive hybridization of rRNAs with antisense rRNA probes (Stewart *et al.*, 2010) recovered from a mixture of DNA obtained from 0.1-, 0.8- and 3.0-μM filters from sampling stations GS265-GS272 (Table 1). Multiple rounds of subtractive hybridization of rRNAs were used to obtain an average of 30 ng of rRNA-subtracted total RNA. In all, 5–10 ng of rRNA-subtracted total RNA was polyadenylated with PolyA Polymerase and subsequently amplified using the MessageAmpII-Bacteria kit (Life Technologies) following the manufacturer's protocol with some modifications. Briefly, amplified rRNA-subtracted RNA was converted to double-stranded cDNA via reverse transcription primed with a modified Oligo(dT) primer containing a promoter sequence for T7 RNA polymerase and a recognition site for the restriction enzyme BpmI (Stewart *et al.*, 2010). cDNA was then subjected to two rounds of *in vitro* transcription at 37 °C for 14 h yielding large quantities (100–200 μg) of single-stranded antisense RNA. Amplified RNA was converted to double-stranded cDNA using the SuperScript III First-Strand Synthesis System (Life Technologies) with priming via random hexamers for the first-strand synthesis and the SuperScript Double-Stranded cDNA synthesis kit (Life Technologies) for the second-strand synthesis. cDNA in the 0.3–3.0-kb size range was purified from agarose gels using the QIAquick Gel Extraction Kit reagents and protocols (Qiagen) and digested with BpmI for 2–3 h at 37 °C to remove poly(A) tails. Following BpmI digestion, cDNA was purified with Ampure XP beads (Beckman Coulter, Brea, CA, USA) and used directly for pyrosequencing. For polyA mRNA transcriptomes, 200 ng of DNase-treated total community RNA was amplified using the MessageAmpII aRNA Amplification kit (Life Technologies) with two rounds of *in vitro* transcription at 37 °C for 14 h with T7 Oligo(dT) priming. Amplified RNA was then converted to double-stranded cDNA using the SuperScript III First-Strand Synthesis System (Life Technologies) with random hexamers for the first-strand synthesis and the SuperScript Double-Stranded cDNA synthesis kit (Life Technologies) for the second-strand synthesis. cDNA in the 0.3–3.0-kb size range was purified from agarose gels using the QIAquick Gel Extraction Kit reagents and protocols (Qiagen), further purified with Ampure XP beads (Beckman Coulter) and used directly for pyrosequencing.

### Reference transcriptome

*Pelagomonas sp*. CCMP1756 was acquired from the CCMP algal collection. Half-liter cultures of axenic *Pelagomonas* were grown in 0.2-μm filtered and autoclaved seawater amended with AQUIL trace metals (Sunda *et al.*, 2005), F/2 vitamins, and filter sterilized nitrogen and phosphorus. Cultures were maintained at 24 °C and 125 μEinstein m^−2^ sec^−1^ with a 14:10 light cycle and bubbling by filter-sterilized air. Two cultures were harvested during exponential growth and nutrient-replete conditions with either nitrate or urea added as a sole nitrogen source. Two cultures were amended with 100 μM NO_3_^−^ and 37.5 μM PO_4_^−^ (N-limited) and 880 μM NO_3_^−^ and 10 μM PO_4_^−^ (P limited). These limitation cultures were harvested when the growth rate began to decline. A final nutrient-replete culture was placed in the dark for 24 h, shifted into full light and then harvested after 2 h. For all harvests, triplicate 50-ml aliquots of culture were collected by centrifugation (1 min, 10 000 *g*) and flash frozen in liquid nitrogen. RNA was purified from filters using the Trizol reagent (Life Technologies), treated with DNase (Qiagen) and cleaned with the RNeasy MinElute Kit (Qiagen). OligoDT primers were used for cDNA synthesis (SuperScript III, Life Technologies) followed by the the SuperScript Double-Stranded cDNA synthesis kit (Life Technologies) for second-strand synthesis. Size-selected cDNA (0.5–1 kb) was purified using the QiaQuick gel extraction kit (Qiagen) and further purified with AMPure (Beckman Coulter). A high-sensitivity DNA Assay chip was used to assess quality (Agilent; Santa Clara, CA, USA). Library construction was conducted according to the manufacturer's directions (ScriptSEQ, Epicenter Technologies, Chicago, IL, USA). Transcriptomes were sequenced on the Illumina HiSeq (Illumina, San Diego, CA, USA). Reads were trimmed of primer sequences and assembled *de novo* using CLC Genomics workbench. Putative open ready frames on the assembled contigs were called using FragGeneScan (Rho *et al.*, 2010). Putative protein sequences were annotated using the hidden markov models and a blastp against PhyloDB 1.02 as described below. *Pelagomonas* RNAseq data have been deposited at the NCBI sequence read archive under accession SRX278429–SRX278432 and SRX278437.

### Sequence annotation

All metagenomic sequence libraries were filtered to remove artifical duplicate reads using CD-hit-454 (Niu *et al.*, 2010). Metatranscriptomic sequences were compared against SILVA to remove 16S rRNA (Pruesse *et al.*, 2007). We also aligned reads against an in-house database of rRNA sequences and whole rRNA operons. All hits with an e-value <10e-10 were considered to be rRNA and were removed from further analysis. The remaining reads were compared with PhyloDB 1.02 in two separate BLAST searches to establish phylogenetic and functional annotation. PhyloDB is a combination of many public protein sequence databases, including KEGG (Kanehisa *et al.*, 2011), IMG (Markowitz *et al.*, 2010), GenBank (Benson *et al.*, 2011), Ensembl (Flicek *et al.*, 2011), several in-house assemblies of algal uniculture transcriptomic sequences, the metagenomic assemblies of SAR86 (Dupont *et al.*, 2012) and HNLC *Prochlorococcus* (Rusch *et al.*, 2010) and the single-cell genomes of SAR324 (Chitsaz *et al.*, 2011) and SAR86 (Dupont *et al.*, 2012). PhyloDB protein sequences (*n*=14 million) come from a wide array of sources, but only proteins directly annotated in KEGG serve as the source of annotations, such as EC and KO. All phylogenetic annotations are generated from the best hit to any protein in PhyloDB. The cutoff used for BLASTing PhyloDB was 1e-5. The significance of read counts between conditions was evaluated using a Chi-squared contingency table test, and *P*-values were calculated for individual species. Multiple testing correction was performed using Benjamini–Hotchberg. Calculations were done using the chisq.test() and p.adjust() functions, respectively, in the stats package in R.

To determine the mean pairwise genetic distance for each community, core marker proteins were first placed into maximum likelihood reference trees as described in Kembel *et al.* (2011). Mean pairwise distances were computed directly from protein placements within these reference trees using the underlying branch lengths. To account for possible effects of sequencing depth, the data sets were bootstrapped to contain the same number of core proteins.

In order to best compare transcriptomes without size fractionation with size-fractionated metagenomes, the size-fractionated metagenomes were combined, with normalization for biomass and bacterial read recovery to reconstruct the original non-size-fractionated metagenome. For the Global Ocean Sampling project, we use a standardized DNA extraction method to allow for comparative genomics (Morgan *et al.*, 2010) and, in this study, DNA yields (mg l^−1^ seawater) for each station (Table 1) correlated with PC and particulate nitrogen (PN) measurements (*r*^2^=0.85 and 0.75, *P*<0.05, respectively). To account for the differences in biomass collected on each filter, we normalized the abundance of an organism based on the amount of DNA extracted from each filter. The normalized proportion *p*_*ij*_ of DNA for bacterial species *i* on filter *j*:


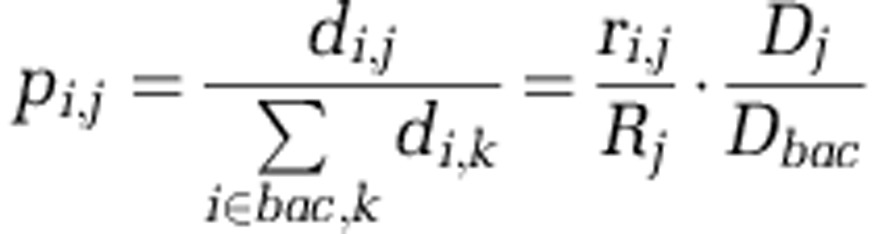


Where *D*_*j*_ is the total DNA in μg on filter *j*; *R*_*j*_ the total reads on filter *j*; *d*_*i*,*j*_ is the DNA of species *i* on filter *j*; and *r*_*i*,*j*_ the number of reads of species *i* on filter *j*. We assume no species read bias, thus *d*_*i*,*j*_/*r*_*i*,*j*_=*D*_*j*_/*R*_*j*_ for all species *i*. Thus *d*_*i*,*j*_=*r*_*i*,*j*_*D*_*j*_/*R*_*j*_ and *D*_*bac*_=∑_*i*∈*bac*,*k*_*r*_*i*,*k*_*D*_*k*_/*R*_*k*_ over all bacterial species *i* and all filters *k*, and ∑_*i*∈*bac*,*k*_*p*_*i*,*k*_=1 for all bacterial proportions. The proportion of a particular bacterial species DNA on a filter is thus the relative number of reads of that species on the filter weighted by the relative contribution of DNA on that filter.

It is important to normalize by filter DNA yield when combining filters for analysis, as this reflects the same result as if the DNA measured on each intervening filter was allowed to pass through and was then collected on a single filter. Both the amount of DNA collected on a filter and the amount of sequencing must be considered from the ratio *D*_*j*_/*R*_*j*_ for filter *j*, which is used to relate the number of reads attributed to a species to the amount of DNA that a species contributed to a filter. For example, consider that a bacterial species has 10, 20 and 40 reads on the 0.1-, 0.8- and 3.0-μm filters, respectively. Each filter has 1000, 4000 and 800 sequenced reads, and 1, 2 and 4 μg DNA total on each filter, respectively. The total amount of DNA contributed by the given species is then (10 × 1/1000)+(20 × 2/4000)+(40 × 4/800)=0.22 μg. If there were 100, 200 and 400 total bacterial reads, respectively, on each filter, then this would yield (100 × 1/1000)+(200 × 2/4000)+(400 × 4/800)=2.2 μg of bacterial DNA (represented by the *D*_*bac*_ term in the equation above) out of the 7 μg of total DNA. The bacterial proportion of the given species would thus be 0.22/2.2=10%. Importantly, the same proportion of 10% bacterial DNA (attributable to the given species in this example) would result if all DNA were to have been collected on a single filter, with no intermediate filtration, and 10% of the bacterial reads were from the given species. Organism-specific transcriptome profiles were examined in MultiExperimentViewer (http://www.tm4.org/mev.html) using the support tree function. To prepare the multiple transcriptomes for comparative RNA-seq analysis, genes with low read counts (<100) were trimmed, and the remaining data were normalized as follows: log_2_ ((*X*+1)/*Y*), where *X* is the read count for a given gene at a given site, while *Y* is the total reads from that site annotated as *Pelagomonas*.

### Phylogenetic placement

To collect the RuBisCO large chain and cytochrome c oxidase sequences, the Pfam (Punta *et al.*, 2012) models PF00016 (RuBisCO_large) and PF00116 (COXI) was searched against an in-house protein database, which was combined from RefSeq (Pruitt *et al.*, 2007) (release 66), JGI (Grigoriev *et al.*, 2012) and MMETSP (Keeling *et al.*, 2014), using HMMER (Eddy, 2011). Similarly, RuBisCO sequences were identified in the metatranscriptome-predicted open reading frames. Representative reference sequences were aligned using MAFFT (Katoh and Standley, 2013). The maximum-likelihood reference tree was inferred with the WAG model (Whelan, and Goldman, 2001) of amino-acid evolution under the gamma model using RAxML (Stamatakis *et al.*, 2008). Finally, the environmental RuBisCO sequences were mapped on the reference tree using pplacer (Matsen *et al.*, 2010).

## Results and Discussion

### Overview of the data set

**Figure 1:**
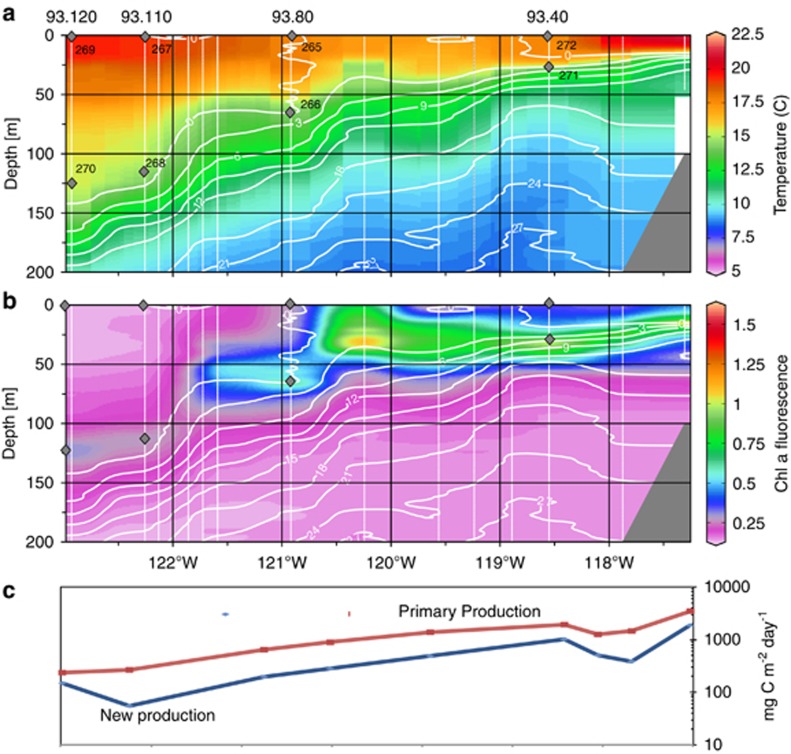
Oceanographic details of the study. (**a**) Cross-section of the CalCOFI line 93 transect in July 2007. Temperature is shown by color gradients while white isobars indicate nitrate concentrations. Diamonds indicate sites of metagenomic and metatranscriptomic sampling. White lines indicate sites of integrated oceanographic measurements. (**b**) As in panel **a**, but color scale indicates chl *a* fluorescence. (**c**) Depth integrated primary production and new production (logarithmic scale).

In July of 2007, 15 stations along a 700-km transect from San Diego, CA out into the subtropical Pacific (Figure 1) were characterized with measurements from a CTD with integrated oxygen, nitrate and chl *a* fluorescence sensors. These highlight a shoaling of the thermocline and nitracline towards the coast with a coincident intensifying of SCM chl *a* content. The SCM of the two mesotrophic sites at stations 93.40 and 93.80 occurred at 25 and 80 m, respectively, while the SCM at the oligotrophic 93.110 and 93.120 were deeper than 100 m. A narrow region of colder, high nitrate, high chl *a* surface water was located around 120.25°W but was not sampled for rate processes and metagenomics. At 12 stations, total PC and PN, phytoplankton pigments, dissolved nutrients, primary production and the uptake of three nitrogen sources were measured at six depths to establish transect-wide gradients. Metagenomic and metatranscriptomic samples were collected from the surface ocean and the SCM at four stations situated within two ‘regimes' of the transect, mesotrophic and oligotrophic. Sequencing was conducted on DNA from the 0.1–0.8-, 0.8–3.0- and 3.0–20-μm size fractions of the microbial community. Sequencing was also performed on cDNA generated from polyadenylated mRNA (polyA mRNA hereafter), which presumably targeted eukaryotes, as well as cDNA generated from rRNA-depleted and *in vitro* polyA mRNAs (Stewart *et al.*, 2010), which targets all RNA (total mRNA hereafter). In both cases, the original RNA was extracted from the same filter, capturing all organisms between 0.1 and 20 μm. In total, between 100 000 and 700 000 sequences for each of the 48 libraries were generated for a total of 13 million reads (Supplementary Table S1).

### Biomass, pigments and production

Overall, communities with a broad range of biomass (Table 1), productivity, nitrogen uptake and light levels were sampled. At all sites, the SCM did not correspond with the PC and PN maxima, which occurred at shallower depths (Supplementary Figure S1). The PC:chl *a* ratios (C:chl *a*) decreased with depth at every metagenomic site, though the gradients were sharper at 93.80, 93.110 and 92.120. The C:Chl a at the SCM of the oligotrophic sites was half that of the mesotrophic sites (Supplementary Figure S1). The mesotrophic sites were also characterized by PC concentrations in excess of 100 μg l^−1^ throughout the euphotic zone, while the PC in the oligotrophic sites rarely exceeded 50 μg l^−1^. The SCM depths at the 93.110 and 93.120 also showed no increase in PC or PN, consistent with their characterization as oligotrophic. Primary production, or the incorporation of CO_2_ into biomass, was also always at maximum at depth, even only 1 km from the coast of California. Despite a shoaling of the euphotic zone, defined here as the depth of 1% incident light, integrated primary production increased dramatically towards the coast (Figure 1). The amount of primary production exported from the euphotic zone, thus escaping regeneration and CO_2_ degassing, is roughly related to the proportion of phytoplankton nitrogen demands satisfied by nitrate (f-ratio), though nitrification can result in an overestimation (Eppley and Peterson, 1979; Yool *et al.*, 2007). To determine *f*-ratios, short-term uptake rates for ammonium, urea and nitrate were determined at the same time as primary production. From stations 93.120 to 93.26, *f-*ratio increased dramatically, resulting in a gradient of depth-integrated carbon export of over 1000 × across the transect (Figure 1).

Phytoplankton community composition was qualitatively assessed using the high-performance liquid chromatography measurements of various accessory pigments along with chl *a*. At each of the surface sites, 19'hexanoylfucoxanthin (19'hex), a pigment diagnostic for haptophytes, was always among the most abundant pigments, along with the *Synechococcus* pigment zeaxanthin. The diatom pigment fucoxanthin was highest, both absolutely and proportionally to chl *a*, at 93.40, the most nearshore station. The dinoflagellate pigment peridinin was not measured due to a lack of a standard. The SCM sites were characterized by increases in nearly all accessory pigments, though 19'butanoylfucoxanthin (19'but) and divinyl chl *a* increased the most relative to chl *a*. The latter suggests an increased abundance of *Prochlorococcus* in each SCM, except for 93.40. Only Pelagophyceae synthesize 19'but however, like haptophytes, also synthesize 19'hex. Relative to surface waters, the 19'but:19'hex in the SCM is 1.5–2 × higher, suggesting that this layer is dominated by pelagophytes as noted in previous pigment surveys (Bidigare and Ondrusek, 1996). All cruise data are available at http://oceaninformatics.ucsd.edu/datazoo/data/ccelter/studies?action=summary&id=1758.

### Metagenomic profiling of microbial communities

The percentage of metagenomic reads that could be annotated declined with the increases in size class; on average 67% of the 0.1–0.8-μm size fraction could be annotated taxonomically compared with 20% of the >3.0-μm fraction (Supplementary Table S1). This drop in annotation, which has been noted in two recently published studies (Ganesh *et al.*, 2014, Satinsky *et al.*, 2014), could be attributed to an abundance of eukaryotic genomes with more non-coding genome regions on the larger filters or a paucity of appropriate eukaryotic reference genomes rather than a lack of appropriate prokaryotic reference sequences. Bacteria composed >80% of the sequences that could be annotated within the smallest size fraction at all sites. Eukaryotes, which accounted for <5% of the reads on the smallest size fraction, contributed 10–50% of the sequences in larger size fractions (Supplementary Figure S2), while bacteria still contributed approximately half of all annotated sequences. Sequences of likely archaeal origin were only abundant in the SCM metagenomes. Sequences of viral origin were surprisingly abundant in the 3–20 μm fraction at all sites, though particularly in the SCM samples where viral sequences accounted for up to 45% of the annotated reads. Notably, a high abundance of viral sequences was observed previously in a single SCM metagenome in the Mediterranean (Ghai *et al.*, 2010), and the results here extend this to multiple geographic locations and large size classes of the community. As viruses are rapidly destroyed by ultraviolet light, the increase of viral abundance with depth and decreasing light might partially explain the trends for the 0.1–0.8-μm fraction yet fails to explain the enrichment in the larger size classes in the surface ocean. Potentially, this observation instead indicates a higher rate of viral replication prior to cell lysis or an increase in cell size-associated lysogenic phage infection.

**Figure 2:**
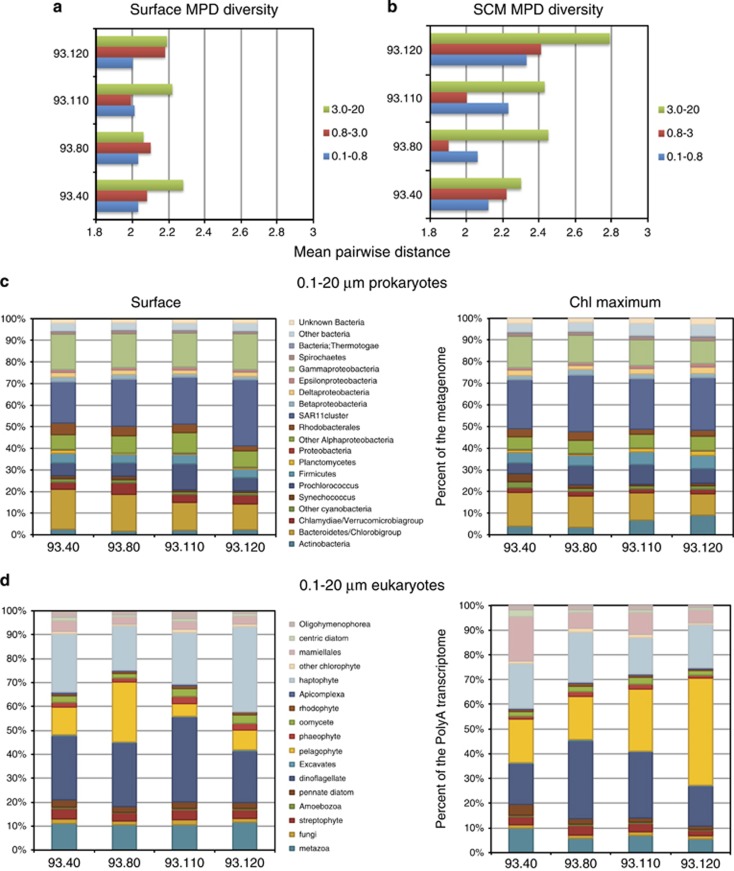
Microbial diversity across the transect. (**a**, **b**) Mean pairwise distance (MPD) of bacterial communities exhibited by each size fraction at each site in the surface (**a**) and SCM (**b**). Bootstraping revealed s.es. to be minimal. Larger values for MPD indicate a greater genetic distance between organisms within a community and likely higher alpha diversity (Kembel *et al.*, 2011). (**c**) Taxonomic breakdown of bacterial communities based on the placement of core marker genes in reference trees. (**d**) Taxonomic breakdown of eukaryotic communities based on BLAST searches against PhyloDB (see Supplementary Methods). For panels **c** and **d**, communities were pooled across size fractions with normalization by the total superkingdom-specific reads within each size fraction.

For community analyses, the prokaryotic, eukaryotic and viral sequences were pooled across all size classes and normalized within superkingdom-specific annotations and, in the case of bacteria, DNA yields from each size fraction. Phyla-level diversity of bacteria and eukaryotes was examined using AMPHORA (Wu and Eisen, 2008) markers and a BLAST search against PhyloDB, respectively, providing a qualitative overview of the biogeography of divisions within these two superkingdoms across environmental gradients (Figure 2). Placement of the AMPHORA marker genes within a bacterial genome reference tree showed that overall mean pairwise distance, a quantitative measure of community diversity (Kembel *et al.*, 2011), was greater in larger size fractions and the SCM sites (Figure 2). Potentially, this reflects mixing between the communities found in the surface euphotic ocean and the dark mesopelagic ocean. Proteobacteria, bacteroidetes and cyanobacteria were the most abundant bacterial phyla altogether, while the dinoflagellates/aveolata, pelagophytes, haptophyes and mamielles accounted for the majority of eukaryotic sequences across the transect. Biogeographical trends include a depth-dependent trade-off of haptophytes and pelagophytes, with the latter dominating the SCM. Similarly, mamielalles, actinobacteria and firmicutes are more abundant in each of the SCM samples while verrucamicrobia are more abundant in the surface ocean (Figure 2). Coastal to open ocean trends include an increased abundance of actinobacteria and pelagophyte at the SCM of oligotrophic sites, and an opposing trend for *Synechococcus* and mamiellales at mesotrophic SCMs.

The number of reads recruited to individual genomes by BLAST analyses were used to identify organisms enriched in size fractions or depths (Supplementary Table S2, *N*=8 for each of 0.1, 0.8 and 3.0, as well as SCM and surface). These confirmed some previous observations related to size-fraction-dependent phylogenetic differentiation (Allen *et al.*, 2012); for example, groups such as the SAR11 clade (*Pelagibacteraceae)*, SAR116 (for example, *Puniceispirillum*), *Prochlorococcus* MED4 and SAR86 were prevalent in the 0.1–0.8-μm fraction in nearly every sample. *Prochlorococcus* also contributed greatly to the bacterial communities in the 0.8–3.0-μm size fraction, as did SAR86, SAR92 (HTCC2207), verrucamicrobia and *Synechococcus*. The 3.0–20-μm bacterial community was enriched in canonical particle-colonizing bacteria from the bacteroidetes (*Chthoniobacter*), verrucamicrobia, flavobacteria (for example, *Lacinutrix, Flavobacterium* ALC-1) and pseudomonads (*Alteromonads*), as well as multiple *Synechococcus* strains. Prokaryotes enriched in the surface ocean included known high-light-adapted *Prochlorococcus* (MED4, MIT9515), organisms most similar to *Coraliomargarita* (verrucamicrobia), the SAR92 bacterium HTCC2207 and several roseobacters (Supplementary Table S2). High-light-adapted *Prochlorococcus* strains were also observed in the SCM, as in previous metagenomic surveys (Ghai *et al.*, 2010, Shi *et al.*, 2011). However, MED4 was mostly confined to the 0.1–0.8-μm size fraction, while more abundant low-light-adapted *Prochlorococcus*, including NATL1A and NATL2A, were found on the 0.8- and 3.0-μm filters, a contrast to previous studies. The SCM sites were enriched in multiple strains of low-light-adapted *Prochlorococcus* with varying abundances (Supplementary Table S2). Other prokaryotes, enriched in each SCM, included organisms similar to the gamma proteobacteria sulfur-oxidizing SUP05 lineage (Walsh *et al.*, 2009) and actinomycetes related to *Frankia* and *Acidomicrobium*. Each of these lineages only composed a small fraction (0.01–0.1%) of the total community and was predominantly found in the 0.1–0.8-μm fraction. It is worth noting that the actinobacteria sequences observed in the SCM are only 30–60% amino-acid identity to the reference (Supplementary Table S2), thus likely are from novel actinobacterial lineages. Potentially, the sulfur oxidizers are ‘seed' populations that bloom with the input of the proper carbon or electron source. The dominant prokaryotic lineages in a hydrothermal vent plume or after an oil spill, for example, originated from the water column where they were <0.1% of the total community (Hazen *et al.*, 2010, Lesniewski *et al.*, 2012).

Even though viral sequences were more prevalent in the largest size fractions at nearly every site, the taxonomic affinity of these sequences varies little, though this could be due to the paucity of viral reference genomes. Sequences from the viral metagenomic libraries from the Chesapeake Bay are among the most abundant in nearly every site and size fraction. Between the surface and SCM samples, a shift in viral taxomic affinity and type occurs. In the SCM samples, caudovirales known to infect *Prochlorococcus* and *Synechococcus* increase in abundance, while in the surface ocean the virus that infects the marine flagellate *Cafeteria roenbergensis* are enriched relative to the SCM (Supplementary Figure S3). Potentially, infection by caudovirales explains the increased enrichment of *Synechococcus* and low-light-adapted *Prochlorococcus* in the larger size fractions of the SCM, due to an increase in cell size with lysogenic or lytic infection. The increased abundance of viral reads in larger size fractions was recently reported for both estuaries (Satinsky *et al.*, 2014) and oxygen minimum zones (Ganesh *et al.*, 2014), suggesting that these are widespread trends.

Within the Eukaryotes, pelagophytes similar to *Pelagomonas* CCMP 1756 (Andersen *et al.*, 1993), as well as the mamielalles *Ostreococcus* and *Micromonas*, were nearly always enriched in the SCM relative to the surface ocean. The one exception was at 93.80, where the *Pelagomonas*-like sequences were slightly more abundant in the surface ocean. This may reflect the doming of isopycnals to the east of this station (Figure 1). Sequences most similar to the non-calcified haptophyte *Phaeocystis* sp. were prevalent in every surface sample, as were sequences most similar to EST sequences from dinoflagellates *Alexadrium* and *Karlodinium*. The phylogenetic profile of the gene sequences from eukaryotes recapitulated some of the trends inferred from the accessory pigments, specifically the abundance of haptophytes in the surface ocean and pelagophytes in the SCM. However, the metagenomic sequencing added additional insight to abundance of the mamielalles in the SCM and the presence of dinoflagellate-like organisms in the surface ocean.

### Metatranscriptomics

The polyA and total mRNA generated transcriptomes contained 0.2–2 and 10–20% rRNA, which was excluded from analysis (Supplementary Table S1, see Methods). In each library type, reads from the non-targeted superkingdom were present (Supplementary Figure S2); 5–10% of the reads from the polyA libraries were of likely bacterial origin, while a remarkable 35–75% of the total RNA libraries associated with eukaryotes. The latter figure sharply contrasts previous metatranscriptomic studies (Gifford *et al.*, 2011; Shi *et al.*, 2011). The discrepancy is likely explained by the use of a 1.6-μm (Shi *et al.*, 2011) or 3.0-μm prefiltration step used in those studies, whereas our transcript pool derives from all cellular organisms from 0.1 to 20-μm.

**Figure 3:**
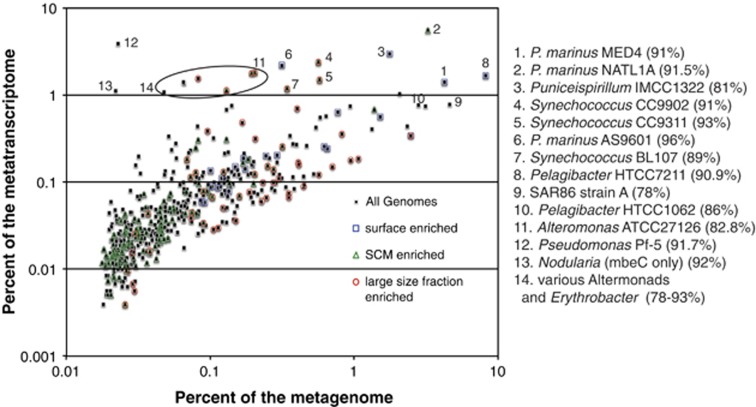
Abundance, habitat and bacterial gene expression. The abundance (in percentage of a pooled metagenome) and gene expression (in percentage of a pooled metatranscriptome) is shown for 500 bacterial genomes that contribute >0.01% of the total metagenome. Genomes enriched 2-fold (surface and SCM) or 50-fold (3.0 μm) in specific environments are indicated, while the number following the genome name indicates the average amino-acid identity of the metagenomic data and the genome.

Roughly 30% of all polyA transcripts could be taxonomically annotated, and at least 100 reads across all samples were associated with one of the 147 available eukaryotic genomes or EST libraries (Supplementary Table S1, Supplementary Figure S4). The taxonomic breakdown of the eukaryotic metatranscriptome and metagenome is well correlated (Supplementary Figure S4). Between 10% and 60% of the total mRNA transcripts could be taxonomically annotated (Supplementary Table S1). On a genome by genome basis, the taxonomic breakdown of eukaryotic transcripts in the total mRNA libraries correlated with observations in the metagenomes and polyA libraries, which were also well correlated (Supplementary Figure S4). In contrast, prokaryotic taxa displayed a much greater divergence of relative representation in metagenomes and metatranscriptomes. Cosmopolitan and abundant organisms, including *Prochlorococcus* MED4, various Pelagibacter and SAR86, were less abundant in the transcriptomes than organisms detected at lower levels in the metagenomes, such as *Synechococcus* (Figure 3). The most transcriptionally active prokaryotes were enriched in the SCM or the larger size fractions (Figure 3). In the SCM, this included the low-light-adapted *Prochlorococcus* (NATL1A, NATL2A, AS9601) and *Synechococcus* sp. CC9311, each of which exhibited very high metatranscriptome versus metagenome ratios. In addition, an assortment of alteromonads and pseudomonads enriched in the 3.0–20-μm fraction contributed greatly to the metatranscriptomes but sparingly to the metagenomes. Such uncoupling between abundance and activity between *Prochlorococccus* strains was recently noted using different methods (Hunt *et al.*, 2013), though results here provide detail on more taxa. Several of the non-dominant but likely active organisms are those readily cultivated, whereas many of the abundant and less active organisms are not generally amenable to cultivation, which led to the moniker ‘uncultivated majority.' These results suggest that the cultivated minority could be disproportionately active and biogeochemically significant, consistent with recent experiments on the growth dynamics and carbon degradation by a single marine *Alteromonas* (Pedler *et al.*, 2014). Note that the relatively modest sequence coverage in this study indicates that truly rare organisms (<0.01%) could not be examined, though potentially the results would be similar. Although our normalization procedure for reconstructing whole-community metagenomes from size-fractionated metagenomes takes into account sequencing depth and DNA yield, we do not correct for genome size. This is due to the lack of a predictive method and sufficient sequencing coverage for taxon-specific genome size estimates. However, we believe our method results in conservative estimates for the transcriptome-to-genome ratios. For example, the larger genome size of *Alteromonas* relative to SAR86 (two related gamma-proteobacteria) means it is more likely to receive relatively higher coverage in a shotgun metagenome. Thus genome size correction would result in smaller-genome/low-transcript organisms such as SAR86 becoming more abundant and larger-genome/high-transcript organisms such as *Alteromonas* becoming less abundant, thus exacerbating the already large differences in transcript-to-DNA ratios.

### Gene expression of specific organisms

Although the low number of transcripts recruited to prokaryotic genomes prevents a robust statistical analysis of gene expression, or even functional genome contents, across the different samples, highly expressed genes for eight of the more highly sampled genomes were examined to gain insight into ecological function (Supplementary Table S3). The abundant though low cDNA:DNA ratio organisms in the *Pelagibacterceae* family predominantly transcribed genes for Na^+^/solute transporters, extracellular solute binding proteins, glycine–betaine-binding cell surface proteins, V-type pyrophosphotases, porins and dicarboxylate transporters, indicating a strategy of low-molecular-weight carbon and osmolyte assimilation as proposed (Steindler *et al.*, 2011). Organisms in the SAR86 clade highly expressed tonB-dependent receptors, cytochrome *c* oxidase, catalase and a V-type pyrophosphatase, potentially reflecting high-molecular-weight carbon uptake and respiration via oxidative phosphorylation (Dupont *et al.*, 2012). Both organisms expressed genes for proteorhodopsin as one of the 20 most abundant transcripts. *Prochlorococcus* and *Synechococcus* predominantly expressed genes involved in photosynthesis, light capture, carbon fixation and outer membrane porins. Genes for sugar uptake and extracellular solute-binding proteins were the most abundant transcripts for the surface ocean clade of SAR324 (Chitsaz *et al.*, 2011). The genomically rare but transcriptionally active *Alteromonad* and *Erythrobacter* expressed a suite of translational proteins, ATP synthase and ribosomal proteins, which suggests a high growth rate relative to other microbes similar to results found at a representative open ocean site (Hunt *et al.*, 2013). Overall, transcriptional profiles for each of the bacterial groups described above suggest a partitioning of ecological roles despite cohabitation, in line with conclusions from recent studies of metagenomic assemblies of uncultivated organisms (Dupont *et al.*, 2012; Iverson *et al.*, 2012) and metatranscriptomes (Gifford *et al.*, 2013; Ottesen *et al.*, 2013).

A large number of transcript reads were associated with the *Nodularia spumigena* CCY9414 genome at 92% nucleotide identity, despite the genome being exceptionally rare in the metaganomic data set (Figure 3). Closer inspection revealed that the reads mapped entirely to one gene on a small (∼1500 bp) contig of the incomplete draft genome. This gene contains a pfam05713 domain and is most similar to the *mobC* gene that codes for the protein MobC from plasmid ColE1, and this contig is most likely a plasmid in *Nodularia.* In plasmid conjugation, proteins participate in two main functions: DNA processing and formation of the cell–cell connection. MobC participates in the DNA processing as an accessory protein to the nicking enzyme (that is, the relaxase). Many plasmids termed ‘mobilizable' have the genes required for nicking but do not have the genes required for full conjugation. They require a second, helper plasmid to make the mating bridge. Thus plasmids like ColE1 can be small, but they are like parasites in that they cannot move themselves without help from other, bigger plasmids. This gene was observed in all the eight total mRNA samples, not in the polyA samples and only rarely in the metagenomes, which may indicate a previously unobserved cosmopolitan distribution for plasmid mobilization.

**Figure 4:**
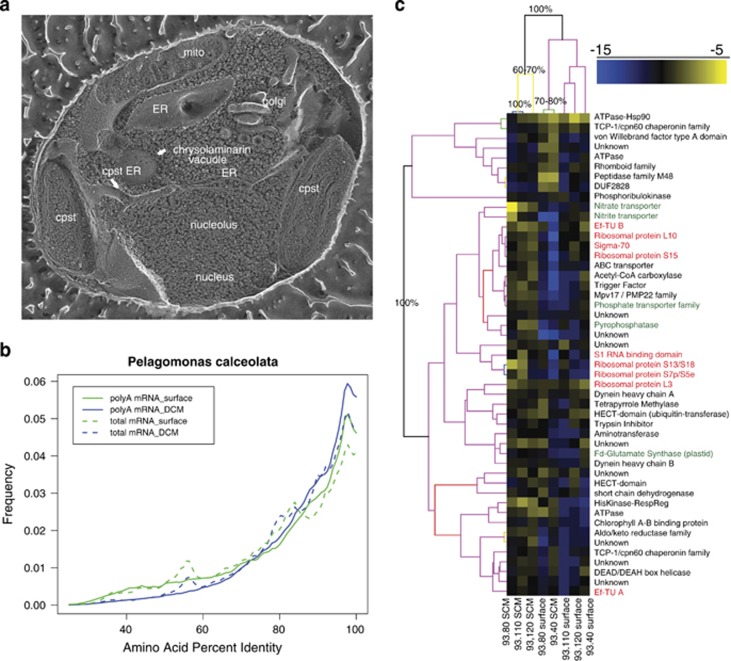
A keystone eukaryote phytoplankton. (**a**) Freeze etch electron micrograph (Heuser, 2011) of *Pelagomonas calceolata* CCMP1756 showing the intracellular structure. (**b**) A smoothed histogram displaying the percentage of identity of metatranscriptomic reads and the CCMP1756 transcriptome generated as part of this study. (**c**) Site-specific gene expression of *Pelagomonas* populations in this study. Both sites and genes were clustered with 1000 bootstraps (support of branchpoints >50% shown). Genes involved in nutrient uptake are indicated in green, while genes involved in translation or growth are shown in red. Relative levels of transcript abundance are indicated with the scale bar.

The polyA transcriptomes were of greater utility for examining gene expression of specific organisms due to the higher depth of coverage. Pelagophyceae sequences only recently were noted to be ubiquitous in the global oceans (Worden *et al.*, 2012), and here were the most abundant eukaryote sequences, thus were targeted for further inspection. Based on analysis of pelagomonas 18S sequences in the metagenomes, we generated a set of transcriptomes from *Pelagomonas calceolata st.* CCMP1756 (Figure 4a) cultures grown under several conditions to provide reference sequences for this organism. BLAST analyses revealed that most Pelegophyte-affiliated environmental transcripts and genome fragments aligned to the CCMP 1756 transcriptome at >90% amino-acid identity (Figure 4b). We further examined the physiology of the CCMP 1756-like populations at each of the sites with a clustering of normalized transcript abundance for the 50 most abundant genes. The SCM from the three most offshore sites grouped together as samples and shared a set of 41 genes expressed at much higher levels relative to the surface populations and the SCM of 93.40 (Figure 4c, bottom 41 rows), where a set of nine genes were expressed at higher levels (Figure 4c, top 9 rows). In the offshore SCM, the most abundant transcripts were those for nitrite and nitrate transporters, carbamoyl-phosphate synthase and a ferredoxin-dependent glutamate synthase (Figure 4c), each of which are required for nitrogen assimilation from nitrate. In addition, multiple ribosome proteins and other genes involved in translation were expressed in the SCM, possibly indicating growth (red genes in Figure 4c). Multiple dynein proteins potentially indicated that these populations are actively motile through flagellar activity and internal axoneme structures (Figure 4c, Supplementary Figure S5). In contrast, the surface and 93.40 SCM *Pelagomonas* communities predominantly expressed stress proteins and chaperones. For the surface sites, this potentially indicates a state of nutrient starvation, consistent with little available inorganic nitrogen. Several transcriptional regulators showed depth-specific expression; one of these genes contains a protein domain (NIT, pfam 08376) involved in nitrate sensing in bacteria. Other regulatory genes with a high level of expression included a gene containing pf00512 (histidine kinase), pf02518 (HisK-associated ATPase) and pf00072 (response regulator) domains. Each of these protein domains is relatively rare in Eukaryotes, suggesting that *Pelagomonas* has adopted a bacteria-like system of transcriptional regulation for nitrogen sensing. The high level of nitrogen-assimilation genes expressed by the pelagophytes in the SCM community is unique among eukaryotes; these organisms contributed >90% of all nitrate transporter transcripts across the entire data set, suggesting that they dominate nitrate uptake and new production across the entire section. This is consistent with a recent stable-isotope-labeling-based flow cytometry study where picoeukaryotic algae dominated nitrate uptake at the SCM of the subtropical Atlantic ocean (Fawcett *et al.*, 2011) and might indicate pelagophytes as a major contributor to such assemblages. Supporting this final assertion are pigment-based analyses from the Sargasso Sea that suggest pelagophytes are a substantial portion of the phytoplankton community (Goericke, 2011).

**Figure 5:**
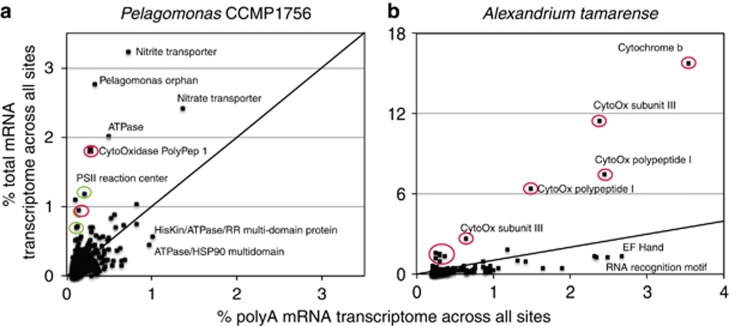
Comparisons of transcriptomes for two eukaryote populations. The representation of genes for *Pelagomonas* (**a**) and *Alexadrium* (**b**) like organisms in polyA and total mRNA transcriptomes is shown as the percentage of transcriptome for each organism. Genes known to be encoded by organelle genomes are indicated in green (chloroplast) and red (mitochondria). Select points that fall off the 1:1 line (black) are annotated.

Qualitatively noting the presence of genes encoded by eukaryote organelle genomes in the total mRNA but not polyA mRNA transcriptomes, we examined the total mRNA:polyA mRNA ratio for all eukaryotic genes with at least 500 reads in the composite polyA library (*n*=105). The average total mRNA:polyA mRNA for the examined set of genes was 0.15, indicating a heavy skew towards polyadenylated gene transcripts. Only 16 genes exhibited ratios >0.5, and many of these are encoded in phytoplankton by either the chloroplast or mitochondrial genome, including cytochrome *c*, cytochrome *b*_6_ and cytochrome *c* oxidase subunit three, and the reaction center proteins for photosystems (*Pelagomonas* and *Alexandrium* annotated transcripts are shown in Figure 5). Organelle genome transcripts are not polyadenylated; therefore enrichment of organelle transcripts within the polyA mRNA transcriptomes has a biological basis. Polyadenylation of eukaryotic organelle transcripts, other than human mitochondrial transcripts, leads to degradation (Schuster *et al.*, 1999, Slomovic *et al.*, 2005), thus these genes might be silenced by nuclear genome-encoded polyadenylases. For example, several parts of the *Alexadrium-*like cytochrome *c* oxidase are highly expressed but likely targeted for degradation through polyadenylation (Figure 5). Potentially, this poises *Alexandrium* to respond rapidly to environmental changes.

**Figure 6:**
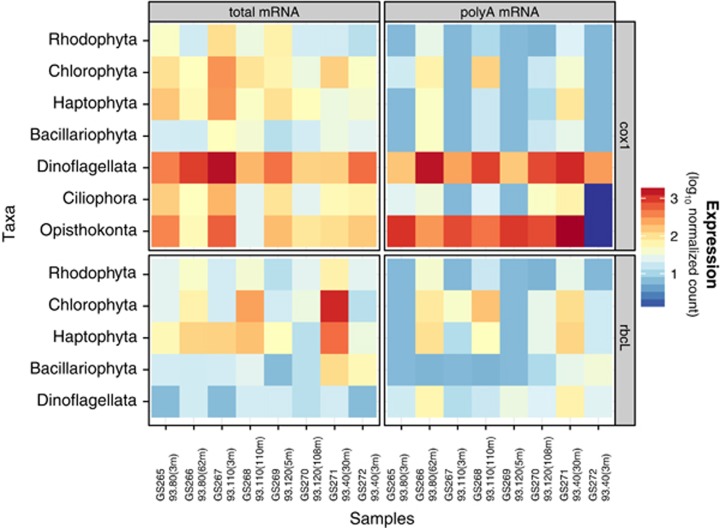
Expression of organelle genes. Heatmap of the expression of polypeptide 1 of cytochrome *c* oxidase (*COX1*) and the large subunit of ribulose-1,5-bisphosphate carboxylase oxygenase (*rbcL*) across the transect. Reads to each gene within the taxonomic categories are normalized for the total number of reads (for a given gene) across the entire transect. Note the log scale (reads (taxonomic lineage *rbcL* at one site)/reads (all *rbcL*)).

**Figure 7:**
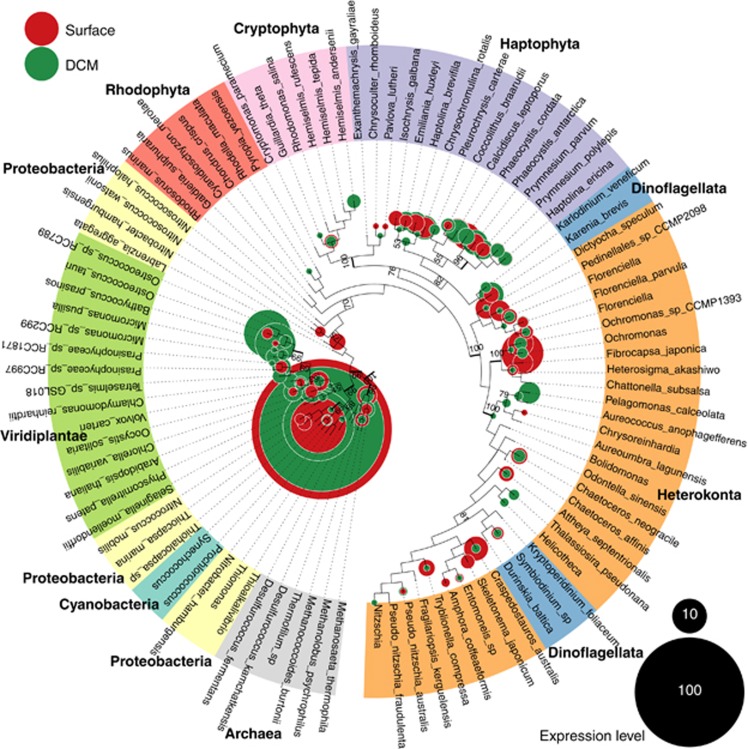
Phylogenetic placement of RuBisCO sequences from surface and SCM samples. RuBisCO large subunit (rbcL) maximum likelihood tree with phylogenetic placement of 2631 metatranscriptome-derived open reading frames. Bootstrap support values are shown when ⩾50%. Thick branches indicate bootstrap support ⩾90%. Bubble sizes are proportion to the average of the rarefied abundances of the surface and SCM samples.

Two of the most important biogeochemical reactions on the planet, carbon fixation and respiration, are commonly quantified during oceanographic surveys without understanding the contribution of individual phylogenetic groups. The expression of cytochrome *c* oxidase 1 and ribulose 1,5-bisphosphate carboxylase/oxygenase (Rubisco) have been examined in both natural systems and organisms and shown to correlate with oxygen consumption or production rates (Corredor *et al.*, 2004; John *et al.*, 2007; Makino *et al.*, 2007; Singtripop *et al.*, 2007). Thus we examined the transcription of polypeptide 1 of cytochrome *c* oxidase (*COX1*, encoded by mitochondrial genomes) and the large subunit of Rubisco (*rbcL*, encoded by plastid genomes) (Figure 6). With few exceptions, the abundance of transcripts, relative to total reads within a particular library, from the total mRNA libraries greatly exceeded those in the polyA mRNA libraries, and the ratio of detection in the two types of libraries varied between the phylogenetic groups and geographic locations. This dramatically illustrates the bias associated with polyA-based transcriptomes. Ultimately, in order to use metatranscriptomics to understand photosynthesis and respiration, both types of library constructions might be required. This approach provides a phylogenetic breakdown of carbon fixation and respiration (Figures 6 and 7). For example, while the high expression levels of *COX1* in opisthokonts, a non-photosynthetic lineage, is consistent with the presumed respiratory metabolism of these organisms, the expression in phytoplankton lineages is striking. Organisms in the dinoflagellata predominantly expressed *COX1* at 10–75-fold higher levels than *rbcL*, which suggests a heterotrophic instead of autotrophic lifestyle. The converse was observed for the heterokonts, bacillophytes and haptophytes, yet these lineages still expressed *COX1* at substantial levels. For example, haptophytes, some of which are likely bacteriovores (Unrein *et al.*, 2014), had nearly equal expression levels of *COX1* and *rbcL* in the surface at 93.80 and 93.110, which may be due to nutrient deprivation making bacterivory the dominant strategy. Many of the phytoplankton lineage organisms, such as diatoms and chrysophytes, generally expressed *rbcL* higher in the surface samples relative to the SCM, though chlorophytes, particularly *Ostreococcus*, and pelagophytes dominated *rbcL* expression in the SCM (Figures 6 and 7). Chlorophytes in the surface ocean appeared to be respiring at much higher rates. Coastal to open ocean trends include an increased contribution of heterokonts (mostly pelagophytes) and diatoms (bacillariophyta) to *rbcL* expression in higher productivity coastal sites. The taxonomic breakdown of prokaryotic photosynthesis and respiration deserves a far expanded treatment than possible here.

### Genome autoecology and possible indicator species

The coupling of metagenomic and oceanographic data sets allows for an examination of the autoecology of species, using genomic enrichment as a proxy. The relative abundance of detected genomes was compared with measurements of nitrate uptake, primary production and chl *a* concentration and examined in the light of overall abundance, size class and gene expression. In a linear regression correlation analysis of the log (percentage of abundance) of 545 bacterial genomes, 40 displayed *r* values >0.7 and *P*-value <0.05 relative to primary production, nitrate uptake or chl *a* concentrations. The majority of these genomes are not abundant within the overall metagenome (average abundance 0.19%), are enriched in the large size fraction (average of 43-fold greater abundance in the larger size fraction) and are transcriptionally active (Supplementary Table S4). Both direct and indirect relationships between oceanographic measurements and genomic enrichment are apparent. An example of a direct relationship is the correlation between primary production, nitrate uptake and the abundance of several strains of marine *Synechococcus*, known carbon-fixing, nitrate-reducing organisms. In an example of indirect interactions, the majority of genomes correlated with chl *a* are from heterotrophic organisms in the bacteroidetes/flavobacteria/sphingobacteria phyla. It has been hypothesized that these organisms are involved in degrading phytoplankton-derived biomass (Teeling *et al.*, 2012), and the correlations observed here support this hypothesis, while extending it to the sphingobacteria. Other bacteria correlated with primary production or chl *a* include the previously mentioned SUP05 sulfur oxidizers (*Ruthia* and *V*e*sicomyosocius*) and the methylotrophs *Methylophilales* and *Methylovorus*, suggesting phytoplankton may produce reduced sulfur compounds and methanol, respectively. Alternatively, SUP05 clade organisms may have been enriched in the SCM due to their abundance in the underlying mesopelagic, rather than a direct relationship with phytoplankton. These correlations potentially identify key indicator organisms, the abundance of which reflects community trophic state. Notably, the low abundance and size fraction distribution of these organisms indicates that prefiltration prior to sample collection or shallow sequencing efforts would exclude these important organisms.

## Conclusions

The integration of size-fractionated metagenomes, metatranscriptomes and oceanographic features and rates provided knowledge of increasing taxonomic specificity with regards to ecological role and community composition. Gradients in light and productivity were associated with shifts in community composition of both prokaryotes and eukaryotes, as were changes in microbial size-class. Both larger size fractions and the SCM exhibited increased phylogenetic diversity. Size-fractionated metagenomics, when combined with a proper normalization technique and metatranscriptomics, can identify relatively rare but transcriptionally active bacteria (Figure 3). In particular, *Synechococcus*, and low abundance gamma-proteobacteria such as *Alteromonas* sp., enriched in the>3.0-μm size fraction, contributed disproportionally to global transcription. This supports an emerging view that some members of cultivated minority could be disproportionately active and biogeochemically significant (Pedler *et al.*, 2014). Curiously, our results suggest that these are also the most responsive to shifts in production, either total or new (Supplementary Table S4). Therefore comparisons of genomic and transcriptomic enrichment with rate measurements suggest previously unappreciated roles for specific taxa.

In each of these types of analyses, comparisons of genomic and transcriptomic enrichment and correlations of genomic enrichment with system productivity and nutrient assimilation, we used either normalized read proportions at the genome or species level or relative enrichment of detected genomes. In both cases, these are measures of the relative abundance of DNA belonging to a particular genome. Further normalization and extrapolation of such data, according to expected genome size, allows estimation of the number of genomes in a sample (Raes *et al.*, 2007), which is thought to more closely represent the number of cells. However, while useful in some cases, such estimates represent a community average (that is, average genome size) and are not suitable for relative comparisons within and between individual species. For analyses aimed at deciphering links between specific microbial taxa and bulk oceanographic rates and properties, species-specific microbial biomass pools would be ideal. Due to the well-known correlation between cell volume and genome size/DNA content (Shuter *et al.*, 1983; Lynch and Walsh, 2007), measurements based on relative DNA content are more likely to correlate with biomass and are therefore well suited for such analyses.

Finally, our results show that pelagophytes, very similar to *Pelagomonas* sp. CCMP1756, appear to be a major constituent of SCM communities and may dominate nitrate uptake and reduction (Figure 4). These results also highlight possible biases in various RNA-sequencing library preparation techniques (Supplementary Figure S3, Figure 6) and suggest the utility of coupled total RNA and polyA transcriptomes to better diagnose both prokaryotic and eukaryotic physiology *in situ*.

## Figures and Tables

**Table 1:** Sample names, sites, coordinates, depth and biomass characteristics for metagenome and metatranscriptome samples

*Sample name*	*CalCOFI site*	*Lat*	*Lon*	*Depth (m)*	*PC* (*μg l^−1^*)	*PN* (*μg l^−1^*)	*DNA_0.1_* (*μg l^−1^*)	*DNA_0.8_* (*μg l^−1^*)	*DNA_3.0_* (*μg l^−1^*)
265	93.80	31.27675	−120.918017	3	238.5	54.6	2.14	0.935	0.375
266	93.80	31.176267	−120.91295	62	121.5	21.3	0.885	1.5	0.54
267	93.110	30.168033	−122.9159	3	44.4	6.9	0.18	0.15	0.08
268	93.110	30.181183	−122.926567	110	6.8	40	0.06	0.14	0.17
269	93.120	30	−124	5	46.5	7.2	0.055	0.02	0.02
270	93.120	30	−124	108	37.5	6.7	0.045	0.04	0.03
271	93.40	32.506083	−118.207867	30	150	28.2	1.38	0.82	1.24
272	93.40	32.513833	−118.2094	3	123.9	19.9	0.64	0.295	0.6

Abbreviations: Lat, latitude; Lon, longitude; PC, particulate carbon; PN, particulate nitrogen.
