# Supplementary material for: Genomes and gene expression across light and productivity gradients in eastern subtropical Pacific microbial communities
Source: ISME J. 2014 Oct 21;9(5):1076–92. doi: 10.1038/ismej.2014.198 (PMC4410273; doi:10.1038/ismej.2014.198)
Supplement: Supplementary Information [file ismej2014198x1.docx]

**Dupont et al, supplemental materials**

**Materials and Methods**

*Primary production*: Photosynthesis (primary production) was estimated from radiolabelled carbon tracer incorporation during 24 h on-deck incubations. Briefly, water samples were collected into 60 ml polycarbonate bottles and inoculated with 10 μCi of ^14^C bicarbonate solution. For each station, one replicate surface sample was filtered immediately to provide time-zero controls. The remaining samples were incubated for 24 h on deck in acrylic incubators screened with blue plus neutral density stage screening to achieve a given percentage of surface irradiance that corresponded to the depth from which they were taken, nominally 100%, 47%, 30%, 16%, 10% and 1%. The depth of each light level was calculated using an inverse analytical radiative transfer model as previous described ([Johnson et al 2002](#_ENREF_6)). Incubator temperatures were maintained by a continuous flow of surface seawater. After incubation, duplicate samples were filtered onto 0.2 μm polycarbonate or Whatman GF/F filters then acidified with 0.5 ml of 0.5 N HCl for 24 h to liberate unincorporated inorganic ^14^C. Ecolume scintillation fluid (7 ml) was added and activity quantified using a Tri-Carb 2900TR liquid scintillation counter (Packard Bioscience, Waltham, MA). Total added ^14^C, was quantified in select inoculated subsamples prior to filtering. Primary production was estimated from the activity of the total and filtered samples using standard calculations ([Barber et al 1996](#_ENREF_1)). When an exact depth match was not present, primary production values were linearly interpolated between nearest neighbor depths to the depth of the interest.

*Nitrogen uptake rates:* Eight 500mL polycarbonate bottles were filled for each of six depths collected 2 hours before dawn. Spikes of 100 nM ^15^NH_4_^+^, ^15^N-urea, or ^15^NO3^-^ (all 99% ^15^N, Sigma) were added to duplicate bottles for each depth and the bottles were transferred to on-deck incubators cooled with surface seawater and shaded to 30% ambient light. After two hours, the entire contents of each bottle was filtered onto a precombusted GF/F (Whatman) filter at low pressure with subsequent storage at-20^o^C in a dessicator. Filters were dried (3 hours at 50^o^C) and packaged in tin for mass spectrometry analysis at the UC Davis Stable isotope facility. Separately, 1L from each of the six depths was filtered gently onto pre-combusted GF/F filters, which were subsequently dried and packaged in tin. The total carbon and total nitrogen captured on these filters were determined at the Scripps Institution of Oceanography analytical facility with a Costech 4010 Elemental combustion analyzer. Uptake rates were determined using the equations described in Dugdale et al ([Dugdale and Goering 1967](#_ENREF_2), [Dugdale and Wilkerson 1986](#_ENREF_3)). In the case where the nutrient concentrations were below the analytical detection limits (100 nM), an ambient concentration of 100nM was assumed.

*Electron microscopy*: Cells in log phase, grown at room temperature at 30 uE (daylight

fluorescent bulbs) on a rotary shaker (125 rev/min) were pelleted; a small aliquot was transferred by toothpick to a cushioning material and dropped onto a liquid He-cooled copper block (4 deg K); the frozen material was transferred to liquid nitrogen and fractured, etched at -80°C for 2 min, and Pt/C rotary replicated as described previously (*). The replicas were examined with a JEOL electron microscope, model JEM

1400, equipped with an AMTV601 digital camera. The images are photographic

negatives; hence, protuberant elements of the fractured/etched surface are most heavily coated with platinum and appear white.

*Pigment measurements*: Two liters of seawater was gently filtered onto a Whatman GF/F filter, which was placed in a cryogenic vial and stored in liquid nitrogen until analysis as described in Goericke and Montoya ([1998](#_ENREF_5)) and Dupont et al. ([2010](#_ENREF_4)).

Table 1: Number of sequences generated for each library and annotation statistics . Schmidt filtering removes duplicate reads. Taxonomic annotation refers to having a match in PhyloDB. Cluster indicates if a sequence is recruited to the GOS protein clusters. KEGG, PFAM, and TIGRFAM indicate the percentage of reads annotated by the indicated families of HMMs.

Table 2: Genomes enriched in the largest size fraction, the SCM, and the surface ocean. No genomes are enriched in the smallest size fraction. All of these genomes are statistically enriched according to multiple methods with p scores less than 0.001 (Chi^2^ test and FDR). Many more genomes are enriched in each environment. The total metagenomic abundance is shown along with total transcriptome abundance. Finally, the percent amino acid identity of the translated transcripts and metagenome reads to each genome is shown.

Table 3: Most expressed genes for several of the most abundant genomes.

Table 4: Genomes whose abundance correlated (r^2^>0.7) with measurements of primary production, nitrate uptake, and chl a. Also shown is the metagenomic abundance of the genomes, its enrichment factor in the largest size fraction, and the CDNA to DNA ratio for each genome.

Figure S1: Depth profiles of particulate carbon, nitrogen, chlorophyll a, and the C:Chl a at each site where metagenomic and metatranscriptomic samples were taken.

Figure S2: Taxonomic breakdown at the domain level for reads that received a taxonomic annotation via BLAST to PhyloDB for each library.

Figure S3: Top panel: Taxonomic breakdown of all annotated reads of putative viral origin for each size class. Bottom panel: Taxonomic breakdown of all annotated reads of putative viral origin for the surface and SCM samples. Only the most abundant taxonomic categories are shown for clarity of legend, thus the histograms do not add up to 100%.

Figure S4: Representation of various eukaryotic (left column) and bacterial (right column) in pooled metagenomes (top four panels) and the two types of transcriptomes. A similar comparison for the two types of transcriptomes is shown in the bottom two panels.


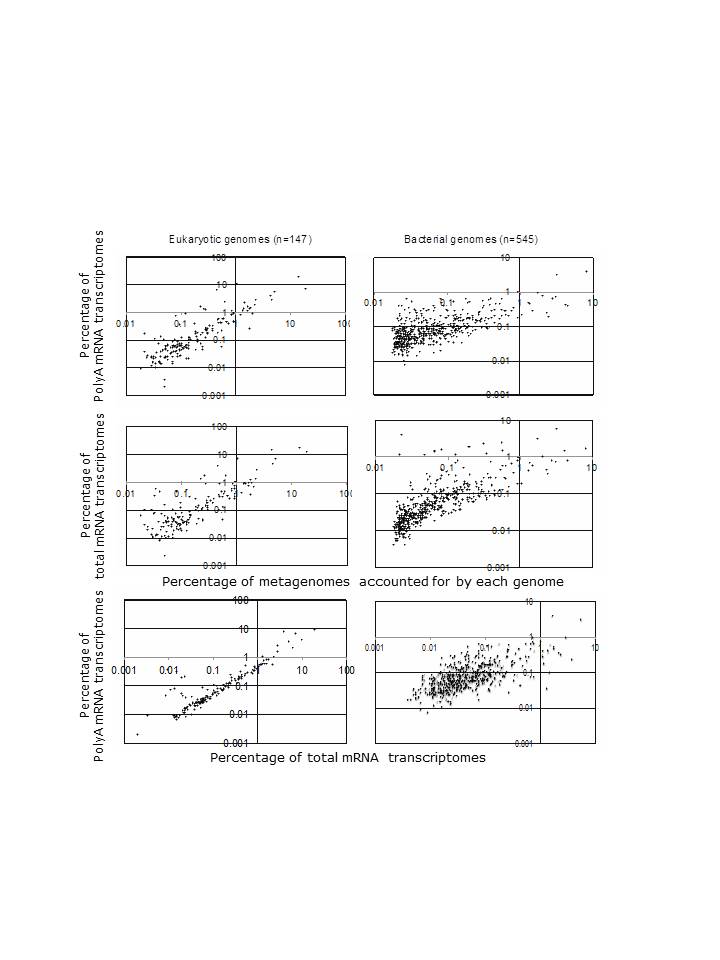


Figure S5: Freeze etch micrographs of the basal body (upper panel) and axoneme (lower) structure in *Pelagomonas calceolate* CCMP1756


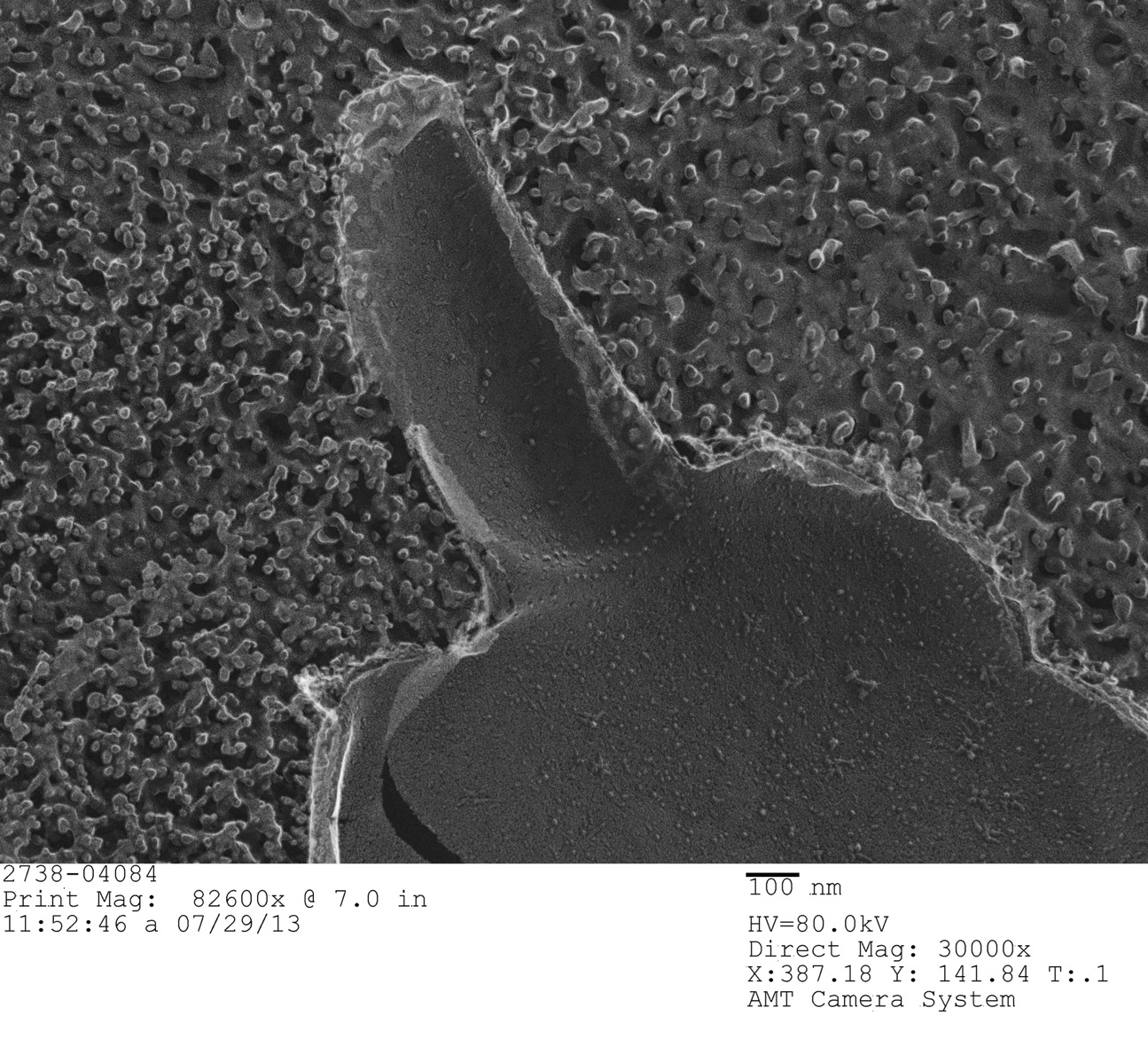


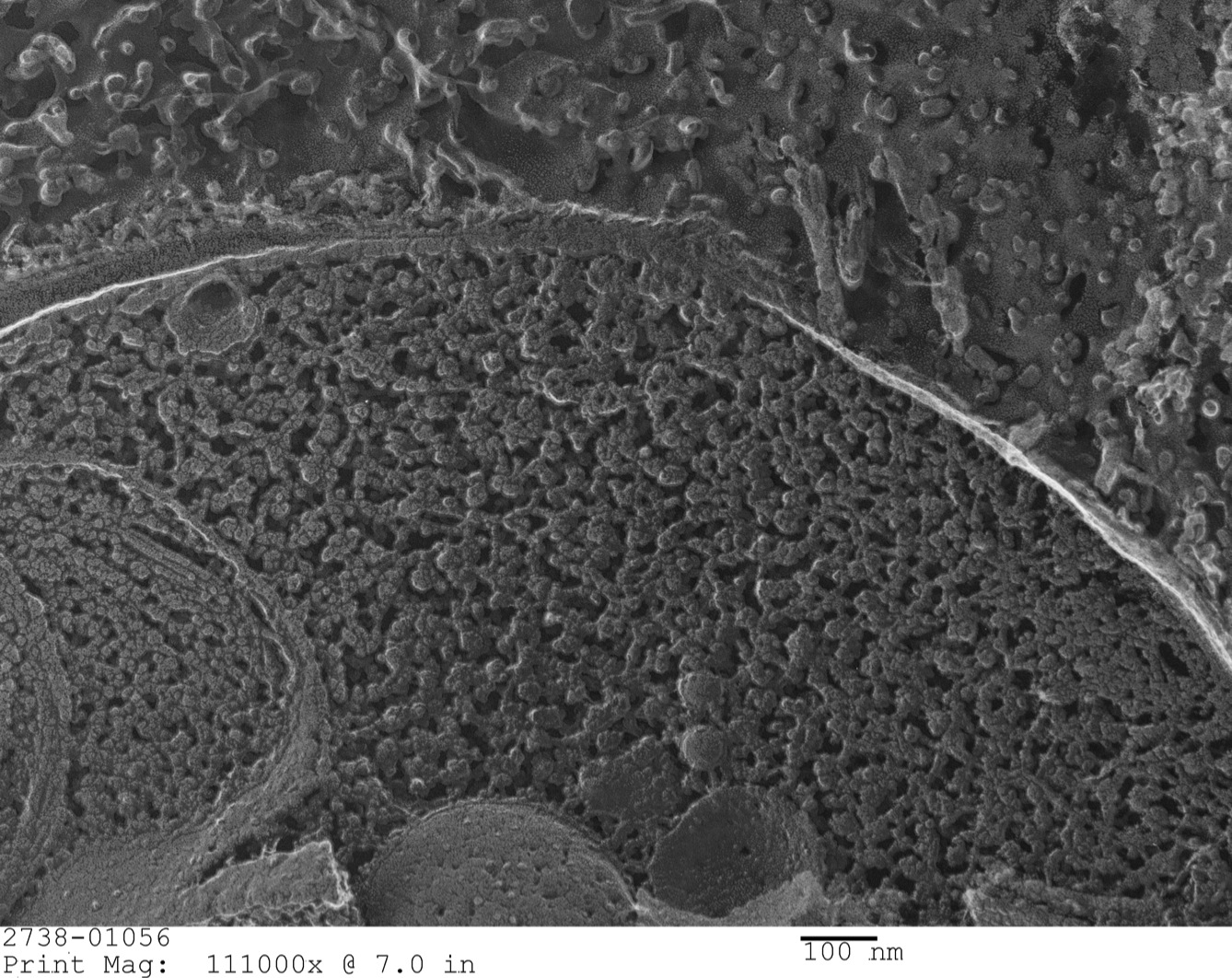


Supplemental References

Barber RT, Sanderson MP, Lindley ST, Chai F, Newton J, Trees CC *et al* (1996). Primary productivity and its regulation in the equatorial Pacific during and following the 1991-1992 El nino. *Deep Sea Research Part II: Topical Studies in Oceanography* **43:** 933-969.

Dugdale RC, Goering JJ (1967). Uptake of new and regenerated forms of nitrogen in primary production. *Limnology and Oceanography* **12:** 196-206.

Dugdale RC, Wilkerson FP (1986). The use of N15 to measure nitrogen uptake in eutrophic oceans: Experimental considerations. *Limnology and Oceanography* **31:** 673-689.

Dupont CL, Buck KN, Palenik B, Barbeau K (2010). Nickel utilization in phytoplankton assemblages from contrasting oceanic regimes. *Deep Sea Research I* **57:** 553-566.

Goericke R, Montoya JP (1998). Estimating the contribution of microalgal taxa to chlorophyll *a* in the field-variations of pigment ratios under nutrient- and light-limited growth. *Marine Ecology Progress Series* **169:** 97-112.

Johnson Z, Bidigare RR, Goericke R, Marra J, Trees C, Barber RT (2002). Photosynthetic physiology and physicochemical forcing in the Arabian Sea, 1995. *Deep Sea Research Part I: Oceanographic Research Papers* **49:** 415-436.

Matsen FA, Kodner RB, Armbrust EV (2010). pplacer: linear time maximum-likelihood and Bayesian phylogenetic placement of sequences onto a fixed reference tree. *BMC bioinformatics* **11:** 538.

Price MN, Dehal PS, Arkin AP (2009). FastTree: computing large minimum evolution trees with profiles instead of a distance matrix. *Mol Biol Evol* **26:** 1641-1650.

Shimodaira H, Hasegawa M (1999). Multiple Comparisons of Log-Likelihoods with Applications to Phylogenetic Inference. *Mol Biol Evol* **16:** 1114.

Sievers F, Wilm A, Dineen D, Gibson TJ, Karplus K, Li W *et al* (2011). Fast, scalable generation of high-quality protein multiple sequence alignments using Clustal Omega. *Molecular systems biology* **7:** 539.

Whelan S, Goldman N (2001). A general empirical model of protein evolution derived from multiple protein families using a maximum-likelihood approach. *Mol Biol Evol* **18:** 691-699.
